# Age and CIRS-G for risk stratification in elderly esophageal squamous cell carcinoma patients receiving definitive chemoradiotherapy

**DOI:** 10.3389/fonc.2026.1898060

**Published:** 2026-08-20

**Authors:** Mingyu Li, Bo Zhang, Jianping Hao, Guoming Song, Ming Zhang, Yunran Hao, Lijun Zhao, Yayuan Wu, Fangming Lu, Qian Zhang

**Affiliations:** Department of Radiation Therapy, The Second Affiliated Hospital of Xingtai Medical College, Xingtai, China

**Keywords:** CIRS-G, definitive chemoradiotherapy, elderly patients, esophageal squamous cell carcinoma, prognosis

## Abstract

**Objective:**

To identify prognostic factors in elderly squamous cell carcinoma (ESCC) patients receiving definitive chemoradiotherapy (dCRT) and construct a prognostic risk stratification model for identifying high-risk subgroups among elderly patients.

**Methods:**

ESCC patients aged ≥ge years receiving dCRT were enrolled. Baseline Cumulative Illness Rating Scale-Geriatrics (CIRS-G), Geriatric Nutrition Risk Index (GNRI), Systemic Immune-Inflammation Index (SII), clinical characteristics and adverse events were collected. Cox regression identified independent prognostic factors, with proportional hazards assessed by Schoenfeld residual tests and 95% confidence intervals validated by 1000 bootstrap resamples. Restricted cubic spline (RCS) curves evaluated potential cutoff values. Survival was analyzed by Kaplan-Meier method and compared by log-rank test.

**Results:**

88 patients were enrolled. Multivariate analysis identified age (HR 1.047, 95% CI 1.000–1.095) and CIRS-G (HR 1.179, 95% CI 1.014–1.376) as independent predictors of OS, whereas GNRI (HR 0.964, 95% CI 0.932–0.995) independently predicted PFS. Schoenfeld tests confirmed proportional hazards (all P > 0.05), with bootstrap resampling supporting coefficient stability. Cutoffs were set at 75 years for age, 7 for CIRS-G, and 98 for GNRI. A prognostic score combining age and CIRS-G stratified patients into low-risk (0–1) and high-risk (2) groups, with high-risk patients showing significantly inferior OS. GNRI ≥98 correlated with prolonged PFS versus <98. The high-risk group had higher neutropenia/leukopenia (15.0% vs. 6.3%) and pneumonitis (5.0% vs. 2.1%), with exclusive thrombocytopenia/anemia (2.5% each).

**Conclusions:**

A composite risk score (age ≥75 years plus CIRS-G ≥7) identified a high-risk subgroup with poor OS. The optimal therapeutic strategy for this subgroup warrants prospective interventional trials.

## 1 Introduction

According to the Global Burden of Disease Study 2023, an estimated 605,000 new esophageal cancer cases and 577,000 related deaths were recorded globally in 2023, ranking it the 6th most common cancer and 6th leading cause of cancer-related mortality (1). The disease exhibits a poor prognosis, with a case-fatality rate approaching 95%. Notably, esophageal squamous cell carcinoma (ESCC) represents approximately 85% of esophageal cancers globally, with the highest age-standardized incidence rates observed across Asian populations (2).

Global population aging has increased the proportion of elderly ESCC. Definitive chemoradiotherapy (dCRT) remains the primary curative strategy for those with poor surgical tolerance, however outcomes remain suboptimal. Since the RTOG 85–01 trial established dCRT as standard care for unresectable locally advanced esophageal cancer, its efficacy has remained disappointingly modest with persistently high rates of local recurrence and distant metastasis. Multiple intensification strategies have been exhaustively explored over the decades. The INT 0122 study escalated both radiation and chemotherapy doses (3), the ARTDECO study pushed radiation to 61.6 Gy using modern intensity-modulated radiation therapy(IMRT) (4), and combination approaches including induction chemotherapy (5), consolidation chemotherapy (6), and immunotherapy maintenance (7) have all been systematically tested. None has demonstrated meaningful survival improvement. In a multicenter prospective study of 298 patients aged 70–85 years, the median overall survival (OS) was only 24.7 months (8).For elderly patients with unresectable esophageal cancer, standard dCRT is associated with substantial treatment-related toxicity. Consequently, in clinical practice, physicians routinely conduct pre-treatment assessments based on age, comorbidities, performance status, and tumor characteristics to estimate prognosis and treatment tolerance. However, no validated, parameter-defined prognostic stratification tool—comparable to the IPI score in lymphoma—currently exists for elderly ESCC patients undergoing dCRT.

The Cumulative Illness Rating Scale for Geriatrics (CIRS-G), which quantifies both the number and severity of comorbidities, has been incorporated into a simplified geriatric assessment (sGA) (9) developed by the Fondazione Italiana Linfomi (FIL) to guide chemotherapy intensity in elderly diffuse large B-cell lymphoma. However, its assessment value in elderly ESCC patients undergoing dCRT remains unclear. Similarly, a simplified frailty score (9) developed by the Nordic Lymphoma Group (NLG) for older lymphoma patients includes the Geriatric Nutritional Risk Index (GNRI), a well-established nutritional risk indicator that has been demonstrated to predict prognosis in elderly esophageal cancer patients, supported by a meta-analysis of 14 retrospective studies comprising 3,981 patients showing significant associations with both overall survival (OS) and progression-free survival (PFS) (10). More recently, systemic inflammatory status has been recognized to modulate the tumor microenvironment, thereby promoting tumor progression (11), and acute exacerbation of chronic diseases,ultimately accelerating mortality in elderly patients (12, 13). The Systemic Immune-Inflammation Index (SII), derived from peripheral neutrophil, lymphocyte, and platelet counts, was identified as an independent prognostic factor in a cohort of 156 ESCC patients receiving dCRT, with elevated SII significantly associated with shorter OS and PFS (14).

This study aims to investigate the prognostic value of CIRS-G, GNRI, SII, and clinical characteristics in elderly ESCC patients undergoing dCRT to determine independent prognostic factors and construct a parameter-defined prognostic stratification tool for identifying high-risk elderly patients.

## 2 Methods

### 2.1 Study population

From January 1, 2023, to December 31, 2024, 88 elderly patients with ESCC were enrolled from the Department of Radiotherapy, Xingtai Medical College Second Affiliated Hospital. Inclusion criteria (1): age ≥65 years (2); pathologically confirmed esophageal squamous cell carcinoma (3); Eastern Cooperative Oncology Group (ECOG) performance status of 1 or less (4); patients who received dCRT as initial treatment without prior surgery, chemotherapy, targeted therapy, immunotherapy, or traditional Chinese medicine (5); absence of hemorrhage or perforation signs in the esophageal lesion prior to dCRT; and (6) complete medical record information and pre-treatment serological laboratory data. The exclusion criteria were (1): total radiotherapy dose <50 Gy or intolerance to concurrent chemotherapy were excluded (2); Clinical TNM stage IV (AJCC 8th edition) and concurrent malignancies (3); coexisting autoimmune diseases; and (4) comorbid life-threatening conditions with an estimated life expectancy of less than 3 months.

Radiotherapy target volumes were delineated as follows: Gross tumor volume (GTV) included the primary tumor and involved lymph nodes; clinical target volume (CTV) encompassed the GTV with a 3–4 cm margin along the longitudinal axis and 0.5cm radially for the primary tumor, and involved lymph nodes were expanded by 0.5cm, with elective nodal regions included; planning target volume (PTV) expanded the CTV by 5–8 mm to account for organ motion and setup uncertainty. Elective nodal irradiation was tailored according to the primary tumor location in accordance with the NCCN guidelines: for upper thoracic esophageal cancer, para-esophageal and supraclavicular lymph node regions were included; for middle thoracic esophageal cancer, para-esophageal lymph nodes were treated; for lower thoracic esophageal cancer, para-esophageal, lesser curvature, splenic, and celiac axis nodal regions were covered. IMRT was delivered at 1.8–2.0 Gy per fraction, five fractions weekly, to a total dose of 50–60 Gy. Organ-atgan-, (OAR) constraints: lung V20 ≤ 30% and mean lung dose ≤ 15 Gy; heart V30 ≤ 40% and mean heart dose ≤ 25 Gy; spinal cord maximum dose ≤ 45 Gy. Concurrent chemotherapy regimens: single-agent cisplatin (20–25 mg/m² intravenously on Days 1–3, every 4 weeks for 2 cycles); S-1 (oteracil potassium, 40–60 mg orally twice daily on Days 1–14, every 3 weeks for 2 cycles); capecitabine (800–1000 mg/m² orally twice daily on Days 1–14, every 3 weeks for 2 cycles).

### 2.2 Data collection

Clinical characteristics were collected prior to dCRT, including sex, age, height, body weight, tumor location, maximum tumor diameter, T stage, N stage, TNM stage (8th edition AJCC), ECOG performance status score, occurrence of radiotherapy interruption, and concurrent chemotherapy regimen. Radiotherapy interruption was defined as a binary variable without stratification by interruption duration. Laboratory data obtained within one week before dCRT comprised serum albumin, neutrophil count, platelet count, and lymphocyte count. The GNRI and SII were calculated using the following formulas:


GNRI=1.489 × serum albumin level (g/L)+41.7 × (actual body weight/ideal body weight),where ideal body weight=22 × height (m)×height (m)

(15),


$$\text{and SII}\left(\times 10^{3}/\text{μL}\right)=\left(\text{neutrophil count}\left(\times 10^{3}/\text{μL}\right)\times \text{platelet count}\left(\times 10^{3}/\text{μL}\right)\right)/\text{lymphocyte count}\left(\times 10^{3}/\text{μL}\right).$$

Treatment response was evaluated by Response Evaluation Criteria in Solid Tumors (RECIST) 4–6 weeks upon completion of treatment.

### 2.3 Assessment of CIRS-G

CIRS-G was assessed based on systematic medical record review and ancillary examinations, evaluating 14 organ systems (cardiac, vascular, hematopoietic, respiratory, upper gastrointestinal, lower gastrointestinal, hepatic, renal, genitourinary, musculoskeletal, neurologic, endocrine/metabolic, psychiatric, and sensory). Each system was scored on a 5-point scale: 0 indicated no impairment, 1 indicated mild impairment that did not require treatment or did not affect daily functioning, 2 indicated moderate impairment that required treatment but was well-controlled, 3 indicated severe impairment that was poorly controlled or required urgent intervention, and 4 indicated extremely severe or life-threatening impairment. The total score ranged from 0 to 56, with higher scores indicating greater comorbidity burden. Two authors (GS and QZ) independently reviewed the hospital medical records and assessed comorbidity for each patient in accordance with the CIRS-G manual. Discrepancies in scoring were resolved by consensus through discussion. The inter-rater reliability for CIRS-G scoring was substantial (Kappa = 0.85, P < 0.001).

### 2.4 Assessment of treatment-related adverse events

Treatment-related adverse events (TRAEs) were assessed and graded according to the Common Terminology Criteria for Adverse Events (CTCAE) version 5.0. Grade ≥r hematologic (neutropenia/leukopenia, thrombocytopenia, anemia), gastrointestinal (nausea/vomiting), and radiation-induced (esophagitis, pneumonitis) toxicities were specifically documented.

Two authors (GS and QZ) independently reviewed the hospital medical records and documented adverse events occurring from the initiation of treatment until 3 months after the completion of dCRT. Treatment-related death was defined as any death occurring within 30 days of treatment completion that was attributable to the study treatment. Discrepancies in grading were resolved by consensus through discussion. The inter-rater reliability for adverse event grading was substantial (Kappa = 0.78, P < 0.001).

### 2.5 Follow-up and endpoints

All patients were followed up through telephone interviews and outpatient visits, with the last follow-up date being March 31, 2026. Patients who were lost to follow-up were censored on the last day they were confirmed alive. OS was defined as the time from dCRT initiation to death from any cause, and PFS as the time to disease progression or death, whichever occurred first.

### 2.6 Statistical analysis

Statistical analyses were performed in R (v4.5.0). Baseline characteristics were summarized using descriptive statistics. CIRS-G, GNRI, SII, age, and tumor length were analyzed as continuous variables in univariate Cox regression analysis. Tumor location (upper vs. middle/lower), T stage (T1–2 vs. T3–4), N stage (N0 vs. N+), TNM stage (I–II vs. III), ECOG performance status (0 vs. 1), radiotherapy interruption (Yes vs. No), and concurrent chemotherapy regimen (cisplatin vs. S-1/capecitabine) were analyzed as dichotomous variables. Variables with P < 0.10 in univariate analysis were assessed for collinearity using variance inflation factor. Variables with variance inflation factor > 5 or tolerance < 0.1 were excluded, and the remaining variables were entered into multivariate Cox regression analysis. Independent prognostic factors were identified accordingly. Two-sided P values < 0.05 were considered statistically significant. Schoenfeld tests confirmed proportional hazards assumptions and non-parametric bootstrap with 1,000 resamples was performed by resampling patients with replacement to assess coefficient stability.

Restricted cubic spline (RCS) analysis was used to explore the relationships between continuous variables and survival outcomes, delineates inflection points, and informs optimal cutoff determination and risk stratification. When no statistically meaningful cutoff value was identified, the cutoff was established based on previously published evidence. Kaplan-Meier survival analysis with log-rank tests was performed for risk stratification.

## 3 Results

### 3.1 Characteristics of patients

No missing data were identified. Baseline characteristics are summarized in **Supplementary Table 1**. The median age was 76 years (interquartile range (IQR), 70–81), with a slight male predominance (51.1%). Tumors were predominantly located in the middle-to-lower esophagus (62.5%), with a median length of 5 cm (IQR, 3.25–7). Most patients had advanced disease (T3–T4, 83.0%; lymph node metastasis, 78.4%; TNM stage III, 63.6%). ECOG performance status was 0 in 35.2% of patients, and radiotherapy interruption occurred in 20.5%. Concurrent chemotherapy was single-agent, with cisplatin in 5 patients (5.7%) and oral fluoropyrimidines (S-1 or capecitabine) in 83 patients (94.3%). The median SII (×10³/μL), GNRI, and CIRS-G score were 608.21 (IQR, 386.23–859.78), 99.87 (IQR, 93.16–107.04), and 7 (IQR, 6–9), respectively. **Supplementary Table 2** presents the frequency distribution of severity scores for the 14 CIRS-G items, and the most common comorbidities were concentrated in the cardiovascular and respiratory systems.Based on RECIST, 71.62% (53/74) of evaluable cases achieved CR/PR. 14 had no post-treatment imaging data and were therefore unevaluable. During a median follow-up of 24 months (95% CI: 21.53–26.7), the median PFS and OS were 16 months (95% CI: 11.79–20.21) and 25 months (95% CI: 16.01–29.99), respectively.

### 3.2 Independent prognostic factors

In the overall cohort, univariate analysis showed that age (P = 0.001), CIRS-G (P = 0.001) and GNRI (P = 0.047) were significantly associated with OS, while PFS was significantly associated with GNRI only (P = 0.004). Multivariate analysis confirmed age (HR = 1.047 per year increase,95% CI:1.000–1.095; P = 0.048) and CIRS-G (HR = 1.179 per score increase,95% CI:1.014–1.376; P = 0.032) as independent prognostic factors for OS (**Table 1**). For PFS, multivariate analysis included age, CIRS-G and GNRI, revealing that only GNRI was an independent protective prognostic factor for PFS (HR = 0.964 per unit increase, 95% CI: 0.932–0.995; P = 0.019) (**Table 2**). Collinearity diagnostics showed no multicollinearity among predictors (VIF 1.087–1.088, tolerance 0.919–0.920).

**Table 1:** Cox regression analysis of clinical variables with OS.

Variables	Univariate analysis		Multivariate analysis	
	HR (95% CI)	P-value	HR (95% CI)	P-value
Age (per 1-year increase)	1.070 (1.028–1.113)	**0.001**	1.047 (1.000–1.095)	**0.048**
Sex				
Female vs. Male	0.746 (0.408–1.373)	0.349		
Tumor location				
Upper vs. middle-lower	0.683 (0.350–1.331)	0.263		
Tumor length (per 1-cm increase)	1.043 (0.953–1.142)	0.356		
T stage				
T3–4 vs. T1–2	1.175 (0.496–2.786)	0.714		
N stage				
N+ vs. N0	0.780 (0.370–1.645)	0.514		
TNM stage				
III vs.I-II	1.054 (0.547–2.030)	0.875		
ECOG				
1 vs. 0	1.241 (0.655–2.351)	0.508		
Radiotherapy interruption				
Yes vs. no	1.035 (0.479–2.237)	0.931		
Concurrent chemotherapy regimen				
cisplatin vs. S-1/capecitabine	0.891 (0.215–3.695)	0.874		
SII (per 1-unit increase)	1.000 (1.000–1.001)	0.172		
GNRI (per 1-unit increase)	0.969 (0.939–1.000)	**0.047**	0.995 (0.962–1.029)	0.756
CIRS-G(per 1-score increase)	1.254 (1.094–1.437)	**0.001**	1.179 (1.014–1.376)	**0.032**

HR, hazard ratio; CI, confidence interval; Bold values indicate statistical significance (P < 0.05). Continuous variables are expressed as HR per specified unit increase. Variables with P < 0.10 in univariate analysis were included in multivariate analysis.

**Table 2:** Cox regression analysis of clinical variables with PFS.

Variables	Univariate analysis		Multivariate analysis	
	HR (95% CI)	P-value	HR (95% CI)	P-value
Age (per 1-year increase)	1.034 (0.995–1.074)	0.089	1.010 (0.967–1.054)	0.658
Sex				
Female vs. Male	1.007 (0.586–1.730)	0.980		
Tumor location				
Upper vs. middle-lower	0.637 (0.370–1.227)	0.197		
Tumor length (per 1-cm increase)				
T stage				
T3–4 vs. T1–2	1.181 (0.553–2.520)	0.667		
N stage				
N+ vs. N0	0.953 (0.476–1.908)	0.891		
TNM stage				
III vs.I–II	1.609 (0.857–3.020)	0.139		
ECOG				
1 vs. 0	1.143 (0.646–2.021)	0.647		
Radiotherapy interruption				
Yes vs. no	0.994 (0.460–1.93)	0.876		
Concurrent chemotherapy regimen				
cisplatin vs. S-1/capecitabine	0.286 (0.040–2.074)	0.216		
SII (per 1-unit increase)	1.000 (1.000–1.001)	0.133		
GNRI (per 1-unit increase)	0.956 (0.928–0.980)	**0.004**	0.964 (0.932–0.995)	**0.019**
CIRS-G (per 1-score increase)	1.116 (0.996–1.251)	0.059	1.069 (0.946–1.207)	0.285

HR, hazard ratio; CI, confidence interval; Bold values indicate statistical significance (P < 0.05). Continuous variables are expressed as HR per specified unit increase. Variables with P < 0.10 in univariate analysis were included in multivariate analysis.

Schoenfeld tests confirmed proportional hazards assumptions for all covariates (all P > 0.05; **Supplementary Figure 1**). Bootstrap validation (B = 1,000) confirmed coefficient stability, with all 95% CIs overlapping original estimates and excluding the null value (**Supplementary Figure 2**).

### 3.3 Risk stratification

**Figure 1:**
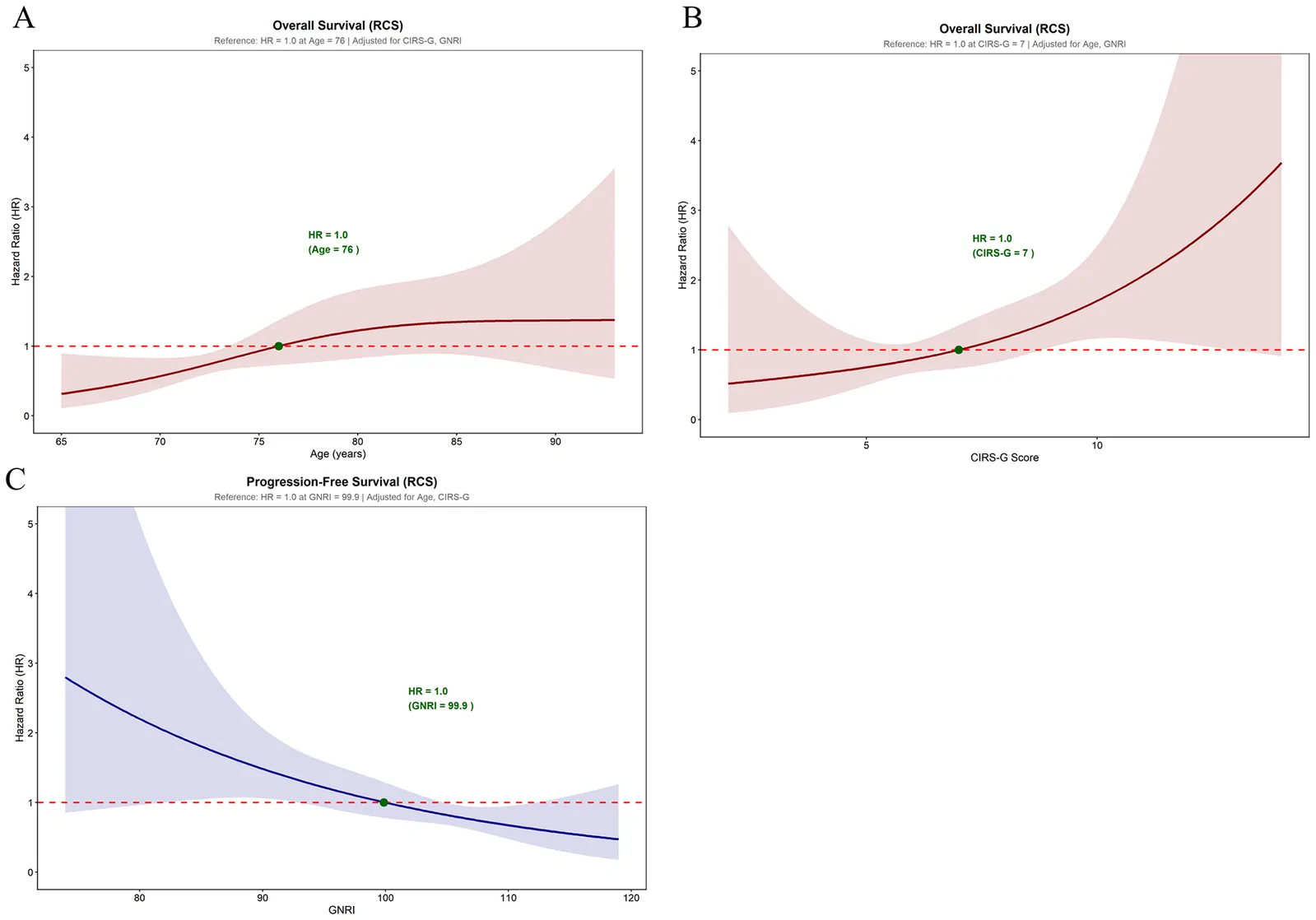
RCS curves showing the dose-response relationships of age, CIRS-G score, and GNRI with survival outcomes. **(A)** Association of age with OS; **(B)** Association of CIRS-G score with OS; **(C)** Association of GNRI with PFS. The solid lines represent the HRS, and the shaded areas indicate the 95% CIs. The reference points (HR = 1.0) are set at age = 76 years, CIRS-G = 7, and GNRI = 99.9, respectively. RCS, Restricted Cubic Spline; HR, hazard ratio; CI, confidence interval; OS, overall survival; PFS, progression-free survival; CIRS-G, Cumulative Illness Rating Scale- Geriatrics; GNRI, Geriatric Nutritional Risk Index.

RCS curves (**Figure 1**) revealed linear and positive associations of age and CIRS-G with OS, and a linear and negative association of GNRI with PFS. No obvious inflection points or plateau effects were observed, indicating continuous prognostic relationships across the entire range of each variable. Accordingly, cutoff values could not be derived from the current dataset and were therefore adopted from published literature: age ≥ 75 years, CIRS-G ≥ 7, and GNRI < 98 (16–18).The rationale for these thresholds is further elaborated in the Discussion.

**Figure 2:**
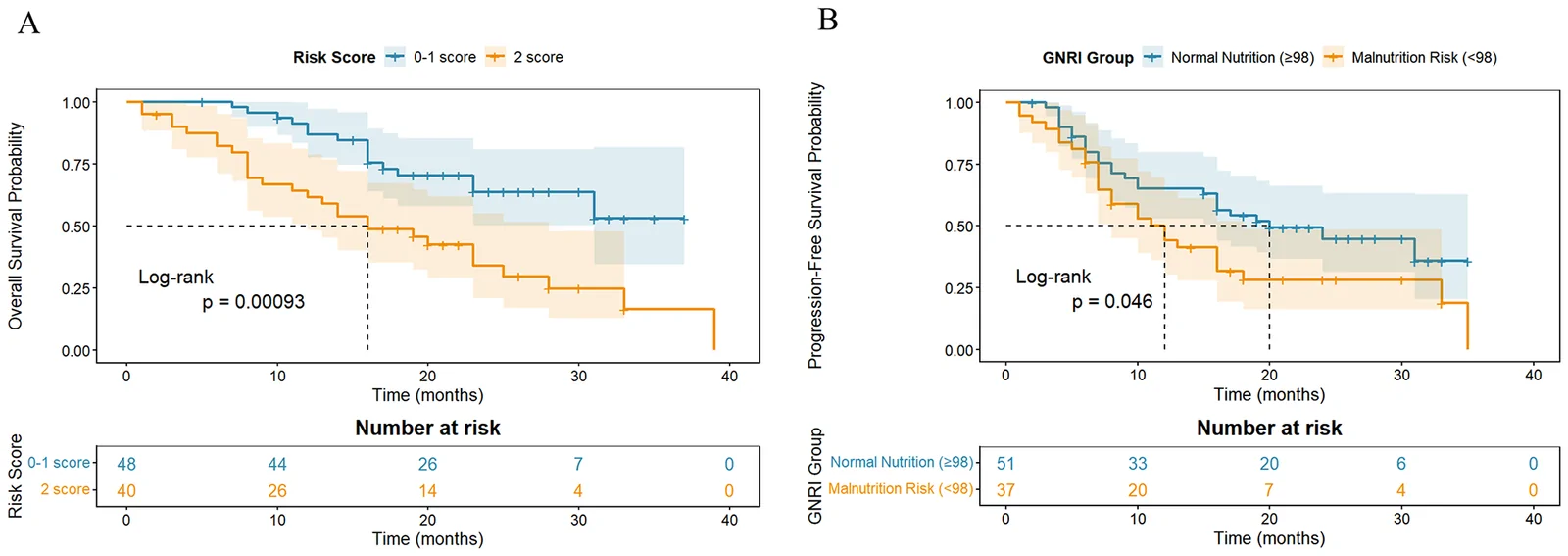
Kaplan-Meier survival curves comparing survival outcomes between different risk groups. **(A)** OS stratified by combined risk score based on age and CIRS-G: 0-1 score (age < 75 years and/or CIRS-G <7) versus 2 score (age ≥75 years and CIRS-G≥ 7), Log- rank p = 0.00093. **(B)** PFS stratified by GNRI: normal nutrition (GNRI ≥98) versus malnutrition risk (GNRI <98), Log-rank p = 0.046. The dashed horizontal line indicates the median survival probability (50%), and the dashed vertical lines indicate the corresponding median survival times. The number at risk table is shown below each curve.

A prognostic score combining age and CIRS-G was constructed to stratify patients into risk groups. Age was dichotomized as <75 years (scored 0) versus ≥75 years (scored 1), and CIRS-G was dichotomized as <7 (scored 0) versus ≥7 (scored 1). Patients with a composite score of 0–1 (age <75 years and/or CIRS-G <7) were assigned to the low-risk group, and those with a score of 2 (both age ≥75 years and CIRS-G ≥7) to the high-risk group. Kaplan-Meier analysis demonstrated that high-risk patients had significantly inferior OS compared with low-risk patients (log-rank P = 0.00093). The median OS was 16.0 months (95% CI, 12.0–28.0) in the high-risk group versus not reached in the low-risk group.Additionally, GNRI ≥98 was associated with prolonged PFS compared with GNRI <98 (log-rank P = 0.046). Kaplan-Meier survival curves are shown in **Figure 2**.

### 3.4 Treatment-related adverse events

The treatment regimen was generally well tolerated, with no treatment-related deaths among the 88 patients. The most common grade ≥ 3 adverse events were neutropenia/leukopenia and radiation esophagitis, each occurring in 10.2% (9/88) of patients. Radiation pneumonitis occurred in 3.4% (3/88), nausea/vomiting in 2.3% (2/88), and thrombocytopenia and anemia each in 1.1% (1/88) (**Supplementary Table 3**).

By risk stratification, the high-risk group exhibited higher rates of neutropenia/leukopenia (15.0% vs. 6.3%) and radiation pneumonitis (5.0% vs. 2.1%), with exclusive occurrence of thrombocytopenia and anemia (2.5% each), whereas radiation esophagitis was more frequent in the low-risk group (12.5% vs. 7.5%).

By GNRI stratification, the GNRItif group demonstrated lower incidences of neutropenia/leukopenia (9.8% vs. 10.8%), radiation esophagitis (9.8% vs. 10.8%), nausea/vomiting (2.0% vs. 2.7%), thrombocytopenia (0% vs. 2.7%), and anemia (0% vs. 2.7%) compared with the GNRI<98 group. Radiation pneumonitis was the exception, occurring only in the GNRIrri group (5.9% vs. 0%).

## 4 Discussion

This retrospective analysis of 88 elderly ESCC patients undergoing dCRT identified three key findings. First, both advanced age and CIRS-G score were independently associated with inferior OS. Second, reduced GNRI independently predicted shortened PFS. Third, patients aged ≥75 years with CIRS-G ≥7 had significantly worse OS than those with either age <75 or CIRS-G <7, while GNRI ≥98 was associated with longer PFS compared to GNRI <98.

### 4.1 Age and CIRS-G as an independent prognostic factor for OS

The prognostic impact of age in elderly ESCC patients treated with dCRT has been demonstrated in large-scale studies, including a SEER database analysis of 869 patients (P = 0.010) (19) and a multicenter Chinese study of 1,013 patients (HR 1.24–1.40 for age ≥75 vs. 65–74 years) (20). However, this association has not been universally observed. A study of 40 elderly patients (≥70 years) found no significant association between age and OS (21), and another of 149 patients (≥75 years) similarly reported age as non-prognostic (22). Several methodological factors may account for these inconsistent findings. Most prior studies treated age as a dichotomous variable in Cox regression analyses, which can obscure its continuous prognostic effect and reduce statistical power (23). The cutoff values employed also varied considerably, potentially contributing to discrepant conclusions. In the present study, age, analyzed as a continuous variable in the Cox regression, emerged as an independent predictor of OS (HR = 1.047, P = 0.048), with each additional year associated with a 4.7% increase in mortality risk.

In elderly cancer patients, greater comorbidity burden (type, number, and severity) independently predicts shorter OS (24). Canoui-Poitrine et al. compared CIRS-G, Charlson Comorbidity Index (CCI), and three CCI-derived indices for 1-year mortality, demonstrating that CIRS-G was the only index with independent prognostic value across metastatic and non-metastatic settings (25). Its independent prognostic significance has been further validated in older patients with primary central nervous system lymphoma, with an HR of 1.037 (P = 0.024) (26). Similarly, elderly patients with limited-stage small cell lung cancer and CIRS-G >5 had worse prognosis (HR = 2.900, P<0.001) (27), and higher CIRS-G scores also predicted early mortality in childhood cancer survivors (28). In this cohort, CIRS-G was further validated as an independent predictor of poor prognosis in elderly ESCC patients, with higher scores conferring a progressively greater hazard.

Comorbidity-driven non-cancer mortality exerts a greater competing risk effect on OS than on PFS (29), particularly in patients of an advanced age. The ELCAPA-19 study enrolled 1,445 cancer patients aged ≥70 years, reporting a non-cancer mortality rate of 17%, which increased to 28% among those without metastasis (30). This proportion further rose to 53% in a cohort of colorectal cancer patients aged >80 years post-surgery (31). These findings suggest that non-cancer mortality increases with advancing age, this trend corroborated by Tan KS et al., who demonstrated a progressive rise in non-cancer mortality with age in lung cancer patients ≥75 years (32). Furthermore, the predominant non-cancer causes of death in these studies originated from comorbidities, particularly cardiovascular diseases. In addition, Müller A et al. found that completing full-dose chemotherapy independently predicts better survival in elderly ESCC patients treated with dCRT (33). Advanced age and high comorbidity increase the risk of treatment discontinuation from toxicities and subsequent poor outcomes. This is another key contributor to the inferior prognosis observed in this vulnerable population.

### 4.2 GNRI: a PFS-specific predictor

GNRI is an established prognostic marker in esophageal cancer, with published studies confirming its association with both OS and PFS (18, 34). In our elderly ESCC cohort receiving dCRT, GNRI independently predicted only PFS, and each oneyicte GNRI increase was associated with a 3.6% risk reduction (HR = 0.964). Low GNRI directly reflects hypoalbuminemia and body weight loss—two parameters that capture the severity of nutritional depletion in cancer patients (35). Serum albumin serves as a substrate essential for maintaining the proliferative capacity of normal tissues during chemoradiotherapy (35). Its deficiency may impair DNA repair mechanisms in normal mucosa, increasing treatment-related toxicity and tissue damage (36). Concurrently, malnutrition-associated immune dysfunction, characterized by decreased lymphocyte counts and impaired cytokine responses, may reduce tumor immunosurveillance during and after treatment, facilitating residual disease progression (37). Additionally, hypoalbuminemia can alter the pharmacokinetics of concurrent chemotherapeutic agents, potentially reducing their efficacy even at standard doses (38). These mechanisms may explain why low GNRI predicts inferior disease control in the short term, though direct evidence remains scarce and whether nutritional intervention improves PFS is unknown.

### 4.3 Composite risk stratification and prognostic model development

RCS curves demonstrated linear, dose-response relationships between age, CIRS-G, GNRI and the outcome, without identifiable thresholds or saturation effects. Therefore, optimal cutoff values could not be derived from the current dataset. For CIRS-G, no universally accepted clinical threshold currently exists. In a study of elderly patients with esophageal cancer receiving dCRT combined with immunotherapy (17), a CIRS-G score ≥7 was used to define severe comorbidity. The Joint Committee of the Japan Gerontological Society proposed in 2017 that the elderly be redefined as those aged 75 years and older (16), reflecting further physiological decline with advancing age. In our cohort, patients aged ≥75 years with CIRS-G ≥7 demonstrated significantly worse prognosis than those <75 years or with CIRS-G <7 (log-rank P = 0.00093), indicating that this subgroup had extremely poor OS. In this study, patients aged <75 years and/or with a CIRS-G score <7 were grouped together as low-risk, as the primary objective was to identify a high-risk subgroup with distinctly poor prognosis for elderly patients, rather than to compare prognoses across all risk categories. A GNRI cutoff of <98 (18), widely recognized and adopted, was defined as indicating high nutritional risk. GNRI < 98 was associated with shorter PFS compared to GNRI ≥98 (log-rank P = 0.046).

### 4.4 Survival outcomes and safety profile in high-risk elderly ESCC

Our institutional practice predominantly employed single-agent oral fluoropyrimidines (94.3%) for elderly patients receiving dCRT. The high-risk subgroup achieved a median OS of 16.0 months (95% CI, 12.0–28.0), with no treatment-related deaths in either group. Compared with the low-risk group, the high-risk group showed higher rates of neutropenia/leukopenia (15.0% vs. 6.3%), pneumonitis (5.0% vs. 2.1%), anemia (2.5% vs. 0%), and thrombocytopenia (2.5% vs. 0%), but lower rates of other toxicities. Adverse events were generally lower or comparable in the GNRI≥98 group versus the GNRI<98 group, except for radiation pneumonitis (5.9% vs. 0.0%). Due to the small number of events, no formal statistical comparisons were performed.

Previously reported retrospective studies have reported 2-year OS rates of 26.0% to 35.5% and median OS times of 8.6 to 15.2 months in elderly patients receiving standard fluorouracil plus cisplatin dCRT (39). Li et al. reported that among elderly ESCC patients aged ≥75 years treated with S-1 plus cisplatin doublet concurrent chemoradiotherapy, the median OS was only 14.3 ± 4.1 months, with substantially higher grade ≥ 3 toxicities compared with patients aged <75 years, including leukopenia (72.0% vs. 48.0%), neutropenia (68.0% vs. 36.0%), anemia (16.0% vs. 4.0%), thrombocytopenia (12.0% vs. 0%), and esophagitis (20.0% vs. 8.0%) (40). Furthermore, Bostel et al. documented treatment-related death during radiotherapy in 2% of elderly ESCC patients receiving full-dose and unmodified chemoradiotherapy in a multi-center cohort (33).

Due to differences in patient selection and study design, a direct comparison is inappropriate even though OS was comparable between these observations of dual-agent concurrent chemoradiotherapy and our results, with higher adverse events reported in the former. Consequently, we cannot conclude that elderly ESCC patients with high-risk features undergoing dCRT would be better suited for single-agent chemotherapy regimens.

### 4.5 Lack of association between SII and survival outcomes

In our cohort, SII failed to reach significance for either PFS or OS. However, dynamic changes in SII during treatment have shown prognostic value in ESCC, and lung adenocarcinoma. This may reflect that post-treatment SII changes are more informative for prognostic assessment. If so, baseline SII would offer limited utility for pre-treatment risk stratification.

### 4.6 Limitations of the study

Despite these strengths, several limitations should be acknowledged when interpreting the results. First, this is a singlescenter retrospective study with a modest sample size of 88 patients, which may limit generalizability and introduce selection bias. Second, the prognostic model was developed and tested within the same cohort, lacking an independent external validation to confirm its reproducibility. Therefore, the results should be interpreted with caution. Third, CIRSd, assessment relies on physician judgment, potentially leading to measurement variability. Additionally, the number of scores 3–4 comorbidities was not separately evaluated, precluding a comparison of the relative prognostic impact of severe comorbidity burden versus the aggregate CIRS-G score on OS. Fourth, chemotherapy regimens were heterogeneous, with the vast majority (94.3%) receiving oral fluoropyrimidines and only 5.7% receiving cisplatin. This marked imbalance precluded stratified or sensitivity analyses by regimen type. Finally, we did not directly compare single-agent versus dual-agent concurrent chemoradiotherapy in this study. As such, we cannot demonstrate that single-agent regimens would achieve comparable survival with lower toxicity in high-risk elderly patients relative to dual-agent dCRT. Consequently, this study should be considered an exploratory study rather than a definitive one.

## 5 Conclusion

In our cohort, both advancing age and CIRS-G emerged as independent prognostic determinants of OS in elderly ESCC patients undergoing dCRT. We developed a simplified prognostic risk stratification model in which the combination of age ≥75 years and CIRS-G ≥7 identifies a high-risk subgroup. Prospective interventional trials are needed to establish whether reduced chemotherapy intensity is appropriate for this high-risk subgroup. Additionally, GNRI <98 was associated with inferior PFS and slightly higher rates of certain toxicities, suggesting a potential role for nutritional intervention in this vulnerable subgroup.

## Data Availability

The original contributions presented in the study are included in the article/**Supplementary Material**. Further inquiries can be directed to the corresponding author.
