# Supplementary material for: Age and CIRS-G for risk stratification in elderly esophageal squamous cell carcinoma patients receiving definitive chemoradiotherapy
Source: Front Oncol. 2026 Aug 20;16:1898060. doi: 10.3389/fonc.2026.1898060 (PMC13537984; doi:10.3389/fonc.2026.1898060)
Supplement: Supplementary file 1 [file Table1.docx]

| Characteristics | No. of Patients |
| --- | --- |
| Clinicopathological features |  |
| Sex, n (%) |  |
| Male | 45（51.1%） |
| Female | 43（48.9%） |
| Tumor location, n (%) |  |
| Upper | 33（37.5%） |
| Middle-lower | 55（62.5%） |
| T stage, n (%) |  |
| T1–T2 | 15（17%） |
| T3–T4 | 73（83%） |
| N stage, n (%) |  |
| N0 | 19（21.6%） |
| N+ | 69（78.4%） |
| TNM stage, n (%) |  |
| I–II | 32（36.4%） |
| III | 56（63.6%） |
| ECOG, n (%) |  |
| 0 | 31（35.2%） |
| 1 | 57（64.8%） |
| Radiotherapy interruption, n (%) |  |
| No | 70（79.5%） |
| Yes | 18（20.5%） |
| Concurrent chemotherapy regimen, n (%) |  |
| cisplatin | 5 (5.7%) |
| S-1/ capecitabine | 83 (94.3%) |
| **Age and tumor characteristics** |  |
| Age (years), median (IQR) | 76（70–81） |
| Tumor length (cm), median (IQR) | 5（3.25–7） |
| **Inflammatory and nutritional indices** |  |
| SII(×10³/μL), median (IQR) | 608.21（386.23–859.78） |
| GNRI, median (IQR) | 99.87（93.16–107.04） |
| **Comorbidity index** |  |
| CIRS-G(score), median (IQR) | 7（6–9） |

Table S1. Baseline characteristics of the study population (N=88).

Note: SII, systemic immune-inflammation index; GNRI, geriatric nutritional risk index; Cumulative Illness Rating Scale-Geriatrics (CIRS-G); IQR, interquartile range. Tumor (T) stage and Node (N) stage were classified according to the 8th edition of the American Joint Committee on Cancer (AJCC) staging system. Tumor-Node-Metastasis (TNM) stage represents the overall stage. Eastern Cooperative Oncology Group (ECOG) performance status was used to assess functional status. Categorical variables are presented as n (%). Continuous variables are expressed as median (IQR).
