# Supplementary material for: Age and CIRS-G for risk stratification in elderly esophageal squamous cell carcinoma patients receiving definitive chemoradiotherapy
Source: Front Oncol. 2026 Aug 20;16:1898060. doi: 10.3389/fonc.2026.1898060 (PMC13537984; doi:10.3389/fonc.2026.1898060)
Supplement: Supplementary file 2 [file Table2.docx]

| **CIRS-G Item** | **0(n)** | **1(n)** | **2(n)** | **3(n)** | **4(n)** |
| --- | --- | --- | --- | --- | --- |
| 1. Heart | 30 | 39 | 6 | 13 | 0 |
| 2. Vessels | 36 | 22 | 16 | 14 | 0 |
| 3. Hematologic | 66 | 19 | 3 | 0 | 0 |
| 4. Respiratory | 24 | 49 | 13 | 2 | 0 |
| 5. ENT (Eyes, Ears, Nose, Throat) | 73 | 15 | 0 | 0 | 0 |
| 6. Upper Gastrointestinal | 39 | 36 | 10 | 3 | 0 |
| 7. Lower Gastrointestinal | 78 | 10 | 0 | 0 | 0 |
| 8. Liver | 37 | 45 | 5 | 1 | 0 |
| 9. Kidneys | 46 | 41 | 0 | 1 | 0 |
| 10. Genitourinary | 67 | 20 | 1 | 0 | 0 |
| 11. Musculoskeletal | 68 | 17 | 3 | 0 | 0 |
| 12. Neurological | 63 | 22 | 3 | 0 | 0 |
| 13. Endocrine/Metabolic | 43 | 24 | 10 | 11 | 0 |
| 14. Psychiatric | 77 | 11 | 0 | 0 | 0 |

Table S2 Frequency distribution of severity scores for the 14 CIRS-G items

Each system was scored on a 5-point scale: 0 = no impairment; 1 = mild impairment that did not require treatment or did not affect daily functioning; 2 = moderate impairment that required treatment but was well-controlled; 3 = severe impairment that was poorly controlled or required urgent intervention; and 4 = extremely severe or life-threatening impairment. Data are shown as the number of participants (n) in each score category.
