# Supplementary material for: Age and CIRS-G for risk stratification in elderly esophageal squamous cell carcinoma patients receiving definitive chemoradiotherapy
Source: Front Oncol. 2026 Aug 20;16:1898060. doi: 10.3389/fonc.2026.1898060 (PMC13537984; doi:10.3389/fonc.2026.1898060)
Supplement: Supplementary file 3 [file Table3.docx]

| Adverse Events | Overall (n=88) | Low-Risk (n=48) | High-Risk (n=40) | GNRI≥98（n=51） | GNRI＜98（n=37） |
| --- | --- | --- | --- | --- | --- |
| Grade ≥3 Neutropenia/Leukopenia | 9 (10.2%) | 3 (6.3%) | 6 (15.0%) | 5 (9.8%) | 4 (10.8%) |
| Grade ≥3 Radiation esophagitis | 9 (10.2%) | 6 (12.5%) | 3 (7.5%) | 5 (9.8%) | 4 (10.8%) |
| Grade ≥3 Radiation pneumonitis | 3 (3.4%) | 1 (2.1%) | 2 (5.0%) | 3 (5.9%) | 0 (0.0%) |
| Grade ≥3 Nausea/vomiting | 2 (2.3%) | 2 (4.2%) | 0 (0.0%) | 1 (2.0%) | 1 (2.7%) |
| Grade ≥3 Thrombocytopenia | 1 (1.1%) | 0 (0.0%) | 1 (2.5%) | 0 (0.0%) | 1 (2.7%) |
| Grade ≥3 Anemia | 1 (1.1%) | 0 (0.0%) | 1 (2.5%) | 0 (0.0%) | 1 (2.7%) |
| Treatment-related death | 0 (0.0%) | 0 (0.0%) | 0 (0.0%) | 0 (0.0%) | 0 (0.0%) |

Table S3. Treatment-Related Adverse Events by Risk Group

Adverse events were graded according to the Common Terminology Criteria for Adverse Events (CTCAE) version 5.0. Low-risk group: score 0-1 (age <75 years and/or CIRS-G <7); high-risk group: score 2 (age ≥75 years and CIRS-G ≥7)
